# Predicting psychiatric drug initiation after craniectomy: a retrospective cohort study

**DOI:** 10.1007/s10143-026-04449-w

**Published:** 2026-09-03

**Authors:** Natalie Simon, Namita Gurung, Nathan Rockley, Katherine Burnett, Rose Ingleton, Soniya Chauhan, Sajjad Saghebdoust, S.M. Saiful Bari, Ahmed Abd Elmohseen, Abdel Rahman Osman, Pouya Behnam, Aimun A.B. Jamjoom

**Affiliations:** 1 Blizard Institute, Faculty of Medicine and Dentistry, Queen Mary University of London, London, UK; 2 Neurosurgery Department, Neurosciences Division, Queen’s Hospital, Barking, Havering, and Redbridge NHS Trust, London, UK

**Keywords:** Craniectomy, Psychiatric morbidity, Risk stratification, Neurorehabilitation, Acquired brain injury

## Abstract

Psychiatric morbidity following craniectomy remains underexplored, with physical disability outcome measures forming the mainstay of follow-up tests used during rehabilitation. There are no predictive models to identify patients at a higher risk of new psychiatric drug initiation. We investigated new psychiatric drug initiation in a mixed-indication cohort following craniectomy and developed a risk stratification model. Our retrospective single-centre study identified 145 patients who underwent a supratentorial craniectomy between 2020 and 2025. Predictor variables were selected based on clinical plausibility, including age, Glasgow Coma Scale (GCS), pupillary response, American Society of Anaesthesiologists (ASA) grade, Index of Multiple Deprivation (IMD) rank, indication for surgery, laterality, and comorbidities. The primary outcome was new psychiatric medication initiation in patients without a pre-existing psychiatric diagnosis. Logistic regression and LASSO penalised modelling were performed. The model was internally validated using bootstrap optimism correction and regression coefficient shrinkage. Across the whole cohort, 33.8% of patients were prescribed psychiatric medication. Of those without a pre-existing psychiatric diagnosis, 18.6% of patients were commenced on new psychiatric medication. Whilst no independent predictors were significant in multivariate analyses, LASSO regression retained higher GCS (coefficient + 0.0585), fewer comorbidities (coefficient − 0.1749), increasing age (coefficient + 0.0109), and a lower ASA (coefficient − 0.0838) as non-zero coefficients under penalisation. Our final model offered limited discrimination (apparent AUC 0.645; Brier score 0.144) with bootstrap internal validation revealing a mean optimism of 0.065 and an optimism-corrected AUC of 0.580 (optimism-corrected Brier score 0.156). We present the first internally validated risk prediction model for new psychiatric drug initiation in a mixed indication craniectomy cohort. Model discrimination was limited, indicating that baseline clinical variables are insufficient to identify patients at increased risk. Prospective studies incorporating gold-standard neuropsychiatric testing are needed before risk stratification could facilitate earlier neuropsychiatric referrals and support more holistic rehabilitation care.

## Background

Craniectomy is a neurosurgical procedure in which part of the skull is removed to reduce raised intracranial pressure and preserve viable brain tissue. It is performed in patients with acquired brain injuries, such as severe traumatic brain injury (TBI), malignant middle cerebral artery (MCA) infarction, and intracerebral haemorrhage, as well as in those with postoperative complications, including infection, cerebral oedema and haemorrhage. Craniectomies are associated with variable outcomes and significant morbidity, with overall high mortality rates [14, 24].

Whilst physical and functional outcomes after craniectomy are well documented [9, 15, 20], neuropsychological and psychiatric morbidity remains underexplored, with affective and cognitive sequelae increasingly recognised in the literature. Cohort studies of patients who underwent decompressive craniectomy (DC) for ischaemic stroke report depressive symptom rates of 26% to 65% [21, 25],whereas studies exploring depression after DC for TBI report a 27% risk at least 1-year post-DC [1]. Whilst there is a paucity of literature examining neuropsychiatric risk in mixed-indication craniectomy cohorts, one small mixed-indication study reported mood disorders in 27% of patients after DC [4].

Disorders of cognition and affect have also been described following DC, with studies reporting deficits in attention, visuospatial perception, memory and executive function [1, 16, 17, 21]. Cognitive dysfunction is common in psychiatric disorders [18], with executive dysfunction significantly linked to emotional regulation in patients with acquired brain injuries [22]. This may be mediated by dysregulation of the top-down control of emotional responses, impairing inhibition and cognitive flexibility and increasing symptoms of rumination and irritability.

Following craniectomy, patients often undergo a cranioplasty, in which the skull defect is replaced with either a synthetic plate or autologous bone. Beyond aesthetic considerations, cranioplasty has been associated with improved motor and neuropsychological function, as well as with clinician-administered mood measures [6, 7, 19]. There is no consensus on the mechanism underlying improvements in mood and neuropsychological function following cranioplasty. One may argue that skull restoration offers patients a greater sense of security and cosmesis, facilitating more confident reintegration into society and reducing feelings of isolation. Changes in neurophysiological parameters, such as increased cerebral blood flow [12] and modulation of cerebrospinal fluid (CSF) hydrodynamics [8, 26], have also been demonstrated, with one study highlighting increased CSF flow velocities and cognitive scores in the same cohort [19]. Whilst this study suggests a potential mechanistic link, it fails to report a formal statistical correlation between physiological measures and cognitive outcomes, indicating the need for further studies exploring causality.

Despite evidence of a significant neuropsychological and psychiatric burden following craniectomy, no predictive models for psychiatric morbidity have been developed, representing an important gap in understanding and risk stratification. We aimed to examine new psychiatric drug initiation in a mixed-indication cohort of patients who underwent a supratentorial craniectomy at our centre, and to explore whether baseline clinical variables, available at the time of craniectomy, can identify patients at a higher risk.

## Methods

### Data collection

We conducted a retrospective single-centre clinical audit and collected data from all adult patients who underwent a supratentorial craniectomy between 1 April 2020 and 1 April 2025. These cases were identified by reviewing paper-based and electronic operative records. The entire population was included in the final analysis with no sampling or exclusions made based on indication.

Clinico-demographic and operative data were collected from electronic patient records (EPR) and our electronic referral portal. This included: Glasgow Coma Scale (GCS) score on presentation, American Society of Anaesthesiologists (ASA) score, pupillary reflex response on presentation, age, laterality and location of craniectomy, indication for craniectomy, cranioplasty status and material used, number of pre-existing medical comorbidities, and Modified Rankin Scale (MRS) at the last follow-up appointment. Psychiatric data were captured from documented past medical history, outpatient letters, and general practitioner (GP) correspondence uploaded to the EPR. The Index of Multiple Deprivation (IMD) rank [23] was obtained from the national government database using patients’ recorded postcodes in the EPR. Psychiatric history was obtained from summary care GP records and the EPR. Follow-up duration was calculated from the date of craniectomy to the date of last recorded clinical follow-up, or to the date of death in patients who died during the study period.

Patients were further classified into two groups based on the indication for craniectomy: the acquired brain injury (ABI) group, including patients with ischaemic and haemorrhagic strokes, traumatic brain injuries, primary brain infections and haemorrhagic space-occupying lesions; and the postoperative complication group, comprising postoperative complications, including postoperative haemorrhage, infection and malignant oedema.

This study was conducted in accordance with clinical governance standards, and formal ethical approval was waived because retrospective anonymised data were collected for analysis.

### Outcome measures

The primary outcome was new psychiatric medication initiation following craniectomy in patients without pre-existing psychiatric diagnoses. This represents a surrogate marker of new-onset psychiatric morbidity. Pre-existing psychiatric diagnoses were recorded separately.

The secondary outcome was prescription of psychiatric medications, regardless of whether they had a formal psychiatric diagnosis.

For patients meeting the primary outcome, the class of psychiatric medication prescribed, and any documented indication were collected from the EPR discharge summary of their inpatient admission during the initial operation and GP summary care records. These medications included any medications with a primary psychiatric indication, as listed in the British National Formulary. Antiepileptic agents were not counted, as these were prescribed frequently for seizure prophylaxis or management. Timing of medication initiation was classified as ‘in-hospital’ where the medication appeared on the craniectomy discharge summary, and as ‘post-discharge’ where it only appeared in subsequent GP records but not on the discharge summary. The exact date of prescription was not consistently recorded. GP records were re-accessed for these patients on 31 July 2026 to determine whether psychiatric medication had been discontinued.

### Statistical analysis and risk modelling

Statistical analyses and figure generation were conducted using Python (version 3.12.3; Python Software Foundation, https://www.python.org), with the following packages: pandas, numpy, scikit-learn, statsmodels and matplotlib.

Statistical significance was defined as *p* < 0.05 for all analyses.

All predictor variables were compared between ABI and postoperative complication groups using either Fisher’s exact test for categorical variables or a Mann-Whitney U test for continuous variables.

To model the primary and secondary outcomes, we ran univariable logistic regression to assess associations between individual predictors and psychiatric medication use. Pre-existing psychiatric diagnosis and psychiatric medication prescription were excluded from the analysis, as the primary outcome is defined as prescription of psychiatric medication without a pre-existing psychiatric diagnosis. MRS was collected as a functional outcome measure but was excluded as a candidate predictor in subsequent analyses because it is assessed only at follow-up, not at the time of craniectomy. Cranioplasty status was similarly excluded as a candidate predictor, as cranioplasty is performed after craniectomy and its status cannot be determined at the time of craniectomy. Age, GCS on presentation, pupillary response on presentation, ASA grade, IMD rank, indication for craniectomy, laterality (coded as left-sided vs. right-sided or bifrontal craniectomy) and number of pre-existing medical comorbidities were selected as the final candidate predictors on grounds of clinical plausibility. Each candidate predictor was entered into a logistic regression model, and unadjusted odds ratios with 95% confidence intervals were calculated. These analyses were run on the entire cohort, the ABI cohort and the postoperative complication cohort.

Following univariate analysis, multivariate logistic regression was performed to assess whether any baseline predictor variables predicted the primary outcome. A complete-case approach was used for the statistical analysis. The estimated adjusted odds ratio and 95% confidence intervals were calculated, and model discrimination was assessed using the receiver operating characteristic curve (AUC).

A notable limitation of multivariate logistic regression is the risk of overfitting in this cohort. To address this limitation, penalised regression was employed. An L1-penalised (LASSO) logistic regression was performed with 5-fold cross-validation, and λ was selected to minimise cross-validated deviance. The rationale was to improve model stability in a small sample by reducing coefficient variance, mitigate overfitting, and improve the events-per-variable (EPV) ratio. Predictors were standardised, and those with non-zero coefficients under the selected λ were retained in the model. All analyses were also run on the secondary outcome measure.

An unpenalised logistic regression was fit using the predictors retained by LASSO, with AUC and Brier scores calculated for the final unpenalised model. Adjusted odds ratios and 95% confidence intervals were calculated from the model. Internal validation was performed through bootstrap resampling using 1000 samples. The model was refitted for each bootstrap sample and applied to the bootstrap sample and original dataset to assess performance. The difference between bootstrap and original dataset performances was defined as the optimism. Optimism-corrected AUC and Brier score estimates were calculated by subtracting the mean optimism across bootstrap samples from the apparent model performance.

The observed and predicted log-odds were used to calculate the calibration slope. The mean optimism bootstrap calibration slope was subtracted from the apparent slope to calculate the optimism-corrected slope. This was used as a global shrinkage factor, and all the regression coefficients were multiplied by this. The model intercept was then refitted.

### Sensitivity analysis: competing mortality

A sensitivity analysis restricting the cohort to patients alive at final follow-up was performed. The same analytical steps were applied to this cohort as the full-cohort: univariate and multivariate logistic regression, LASSO variable selection, unpenalised model refitting using only LASSO-selected predictors, bootstrap internal validation, and shrinkage coefficient adjustment.

## Results

### Cohort characteristics

A total of 145 patients were identified as having undergone a supratentorial craniectomy at our centre over the five-year study period (Table 1). The mean age was 51.7 years (SD 13.4), median GCS on presentation was 10 (IQR 7–14), and median ASA was 3 (IQR 3–4). At the time of analysis, mortality was 31.0% (45/145). Most patients had an ABI (68.3%, 99/145), with 32.4% of the entire cohort comprising haemorrhagic strokes, 19.3% ischaemic strokes and 9.0% TBI. Just under a third of the cohort (31.7%) had a craniectomy secondary to a postoperative complication (including postoperative haemorrhage, infection, or malignant oedema). Of the total cohort, 49 (33.8%) patients were prescribed psychiatric medication, whilst 30 (20.7%) had a pre-existing psychiatric diagnosis. Not all patients with a psychiatric diagnosis recorded on EPR were prescribed psychiatric medication.

In comparison to the ABI cohort, the postoperative complication group presented with a more favourable GCS at admission (median GCS 15 vs. 9, *p* < 0.0001), lower ASA grade (median 3 vs. 4, *p* < 0.0001) and a better functional outcome at follow-up (median MRS 4 vs. 5, *p* = 0.013). There was no difference in comorbidity burden between the groups (median number of comorbidities 2 vs. 1, *p* = 0.506) (Table 2).

**Image 1:**
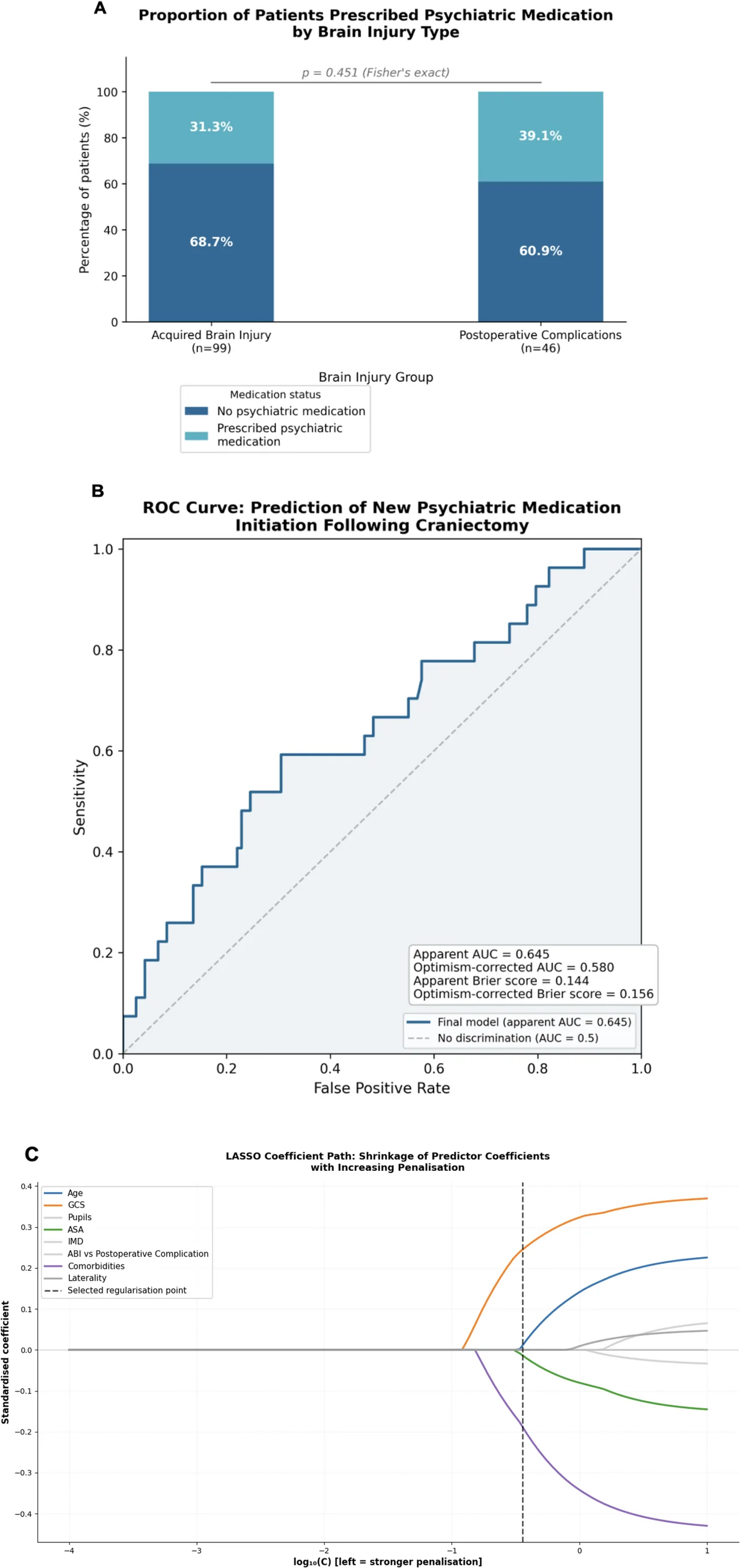
**A**: Psychiatric medication percentage by brain injury type. Bar chart showing the proportion of patients prescribed psychiatric medication by brain injury type. These statistics were compared using Fisher’s exact test. **B**: Receiver operating characteristic (ROC) curve demonstrating model discrimination for the prediction of new psychiatric medication initiation following craniectomy. ROC curve for the shrinkage-corrected prediction model for new psychiatric medication initiation following craniectomy. The model was derived using unpenalised logistic regression on LASSO-retained predictors (GCS, comorbidity burden, ASA and age). **C**: LASSO coefficient path analysis. Each line represents one candidate predictor’s standardised coefficient. Lines above 0 indicate a positive coefficient with psychiatric medication initiation and lines below 0 indicate a negative coefficient. The dashed vertical line is the selected regularisation point chosen by 5-fold cross-validation. Only GCS, comorbidities, ASA and age retain non-zero coefficients

The percentage of patients prescribed psychiatric medications was 39.1% in the postoperative complication group and 31.3% in the ABI group (*p* = 0.451), whilst new psychiatric medication initiation was 21.7% and 17.2% respectively (*p* = 0.502) (Image 1 A).

Follow-up duration was variable across the cohort (median 196 days, IQR 53–599). In patients who died during the study period, the median interval from craniectomy to death was 36 days (IQR 4–76), with just under half (46%) of these deaths occurring within 30 days of surgery. Among survivors, the median follow-up was 379 days (IQR 106–737). Follow-up duration was not available for 2 patients who did not attend scheduled post-operative clinic appointments.

The median interval from craniectomy to cranioplasty in those who had one was 307 days (IQR 130–554). Survivors without a cranioplasty had considerably shorter follow-up: median 181 days (IQR 76–523) vs. 596 days (IQR 385–839) and a greater burden of dependence: MRS 4 (IQR 2–5) vs. 1 (IQR 1–4).

### New psychiatric medication prescriptions: medication classes and clinical characterisation

Among 27 patients who were prescribed psychiatric medications without a psychiatric diagnosis pre-craniectomy, the distribution was: 18 (67%) selective serotonin reuptake inhibitors (citalopram, sertraline, or fluoxetine), 4 (15%) mirtazapine, 3 (11%) tricyclic antidepressants and 2 (7%) atypical antipsychotics. All four mirtazapine prescriptions were at 30 mg daily, a dose used in the management of depression. Three patients received tricyclic antidepressants: amitriptyline 10 mg, amitriptyline 50 mg and nortriptyline 10 mg daily. Seven patients (26%) had a formal indication recorded at the time of prescription (depression in all cases), whilst 20 (74%) were unspecified on GP summary care records.

GP records for the 27 patients meeting the primary outcome were reviewed on 31 July 2026 (final data review timepoint) to determine whether psychiatric medication had been discontinued. Six patients in this cohort had passed away during the study period (prior to 1 April 2025). All 21 survivors remained on the prescribed psychiatric medication at the final review (31 July 2026). This is consistent with chronic psychiatric disorder management rather than acute delirium. Additionally, medications were started in-hospital (during the craniectomy admission) for 14 (52%) patients and following discharge for 13 (48%), suggesting psychiatric symptoms were recognised at different postoperative timepoints post-craniectomy.

### Predictors of psychiatric medication use after craniectomy

**Image 2:**
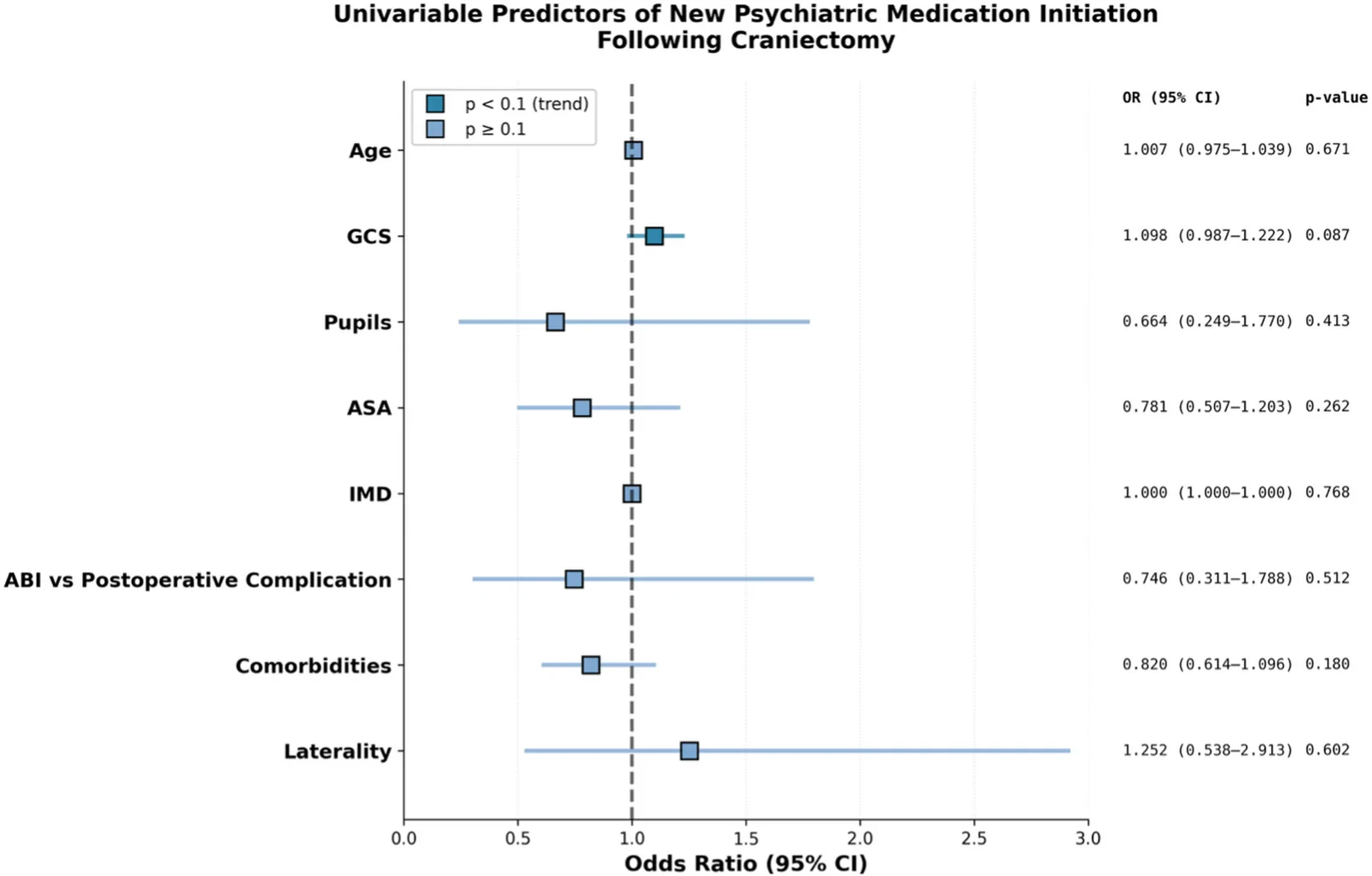
A forest plot displaying univariate predictors of psychiatric medication initiation. Forest plot of unadjusted odds ratios (OR) with 95% confidence intervals (CI) for univariable logistic regression of candidate predictors of new psychiatric medication initiation following craniectomy. The null (OR=1.0) is displayed with the vertical line

The primary outcome was met in 27 patients (18.6%) and was defined as initiation of psychiatric medication in patients without a pre-existing psychiatric diagnosis. In univariate logistic regression, no single candidate predictor for our primary outcome measure reached statistical significance in any group (Image 2). When analyses were repeated for the individual acquired and postoperative complication cohorts, the findings were similar. Given the modest number of primary outcome events and likely confounding among the predictor variables, we reasoned that these predictors may exert effects after adjustment for other covariates. Therefore, to assess the combined predictive power of candidate predictors, a multivariable logistic regression was run on the entire cohort (Table 3). Again, no independent predictor reached statistical significance. Multivariate regression model discrimination was modest (AUC 0.645). The EPV for the whole cohort multivariate model was 3.4, therefore, to mitigate overfitting a LASSO regression was performed prior to refitting the logistic model (Table 4). Under penalisation, only four predictors retained non-zero coefficients: GCS on presentation, number of pre-existing comorbidities, age and ASA. GCS and age were retained with positive coefficients, and ASA and comorbidity burden had negative coefficients, reflecting the direction of penalised estimates. These should not be regarded as statistically significant associations as no candidate predictor reached a p-value of below 0.05 in univariate or multivariate analyses.

### Sensitivity analysis: competing mortality

To establish if mortality confounds the relationship between baseline predictor variables and new onset psychiatric medication prescription, a sensitivity analysis was performed on only patients that survived to the last follow-up (*n* = 100; 69% of the full cohort). These patients had a mean age of 50.1 years (SD 13.3), median GCS on presentation of 11 (IQR 7–14) and median ASA grade of 3 (IQR 2–4). 21 patients (21%) were started on new psychiatric medication without a pre-existing psychiatric diagnosis, which was comparable to the rate observed in the full cohort (18.6%).

Both univariate and multivariate analyses revealed no significant predictors of new psychiatric medication initiation. LASSO regression retained only age (coefficient + 0.0899) and comorbidities (coefficient − 0.1638) as non-zero predictors. GCS, ASA, pupillary response, IMD, laterality, and indication for surgery had no discriminative value after penalisation.

The unpenalised model was refitted using only age and comorbidities, showing apparent discrimination of AUC 0.627 (Brier score 0.155). Bootstrap internal validation (using 1000 resamples) on the alive-only cohort resulted in an optimism-corrected AUC of 0.582, with a mean AUC optimism of 0.045. This is comparable to the full-cohort (optimism-corrected AUC: 0.580; mean AUC optimism: 0.065). The optimism-corrected Brier score was 0.165 (mean Brier optimism: -0.010; shrinkage factor: 0.762).

The final shrunken model was logit(p) = -2.2700 + 0.0209(Age) − 0.2026(Comorbidities), with p representing the probability of new psychiatric medication initiation following craniectomy.

### Final prediction model for being started on psychiatric medications following a craniectomy

For the primary outcome measure, non-zero penalised coefficients were obtained for the model involving the whole cohort, including GCS, ASA, number of pre-existing comorbidities and age. These four predictor variables were used to fit an unpenalised logistic regression.

In the refitted unpenalised model, no individual predictor reached statistical significance after adjustment. The adjusted effects showed point estimates above the null for GCS (aOR 1.093, *p* = 0.133) and age (aOR 1.017, *p* = 0.349), and point estimates below the null for comorbidity burden (aOR 0.766, *p* = 0.087) and ASA score at surgery (aOR 0.880, *p* = 0.602). The apparent model had an AUC of 0.645 and a Brier score of 0.144 (Image 1B), indicating limited predictive capability relative to baseline risk.

Internal validation was performed using 1000 samples. The apparent AUC of 0.645 reduced to 0.580 after optimism correction (mean optimism in AUC was 0.0647) and the optimism-corrected Brier score was 0.156, increasing from 0.144 (mean optimism in Brier score was − 0.0119). A shrinkage-adjusted prediction model was also run. The apparent calibration slope was 1.000, and the optimism-corrected slope was 0.656. This was used as a uniform shrinkage factor. The final shrunken model was logit(p) = -2.4103 + 0.0109(Age) + 0.0585(GCS) − 0.0838(ASA) − 0.1749(Comorbidities), with p representing the probability of new psychiatric medication initiation following craniectomy. The LASSO coefficient path analysis (Image 1 C) showed progressive shrinkage of predictor coefficients with increasing penalisation.

**Table 1:** Summary statistics for the complete analysed cohort

Variable	Value
Demographics Age, years – mean (SD) IMD rank (out of 33,755) – median (IQR)	51.7 (13.4) 15,083 (8,755 − 20,711)
Presentation GCS – median (IQR) Pupillary response – n (%) ASA grade – median (IQR)	10 (7–14) 119 (82.1) 3 (3–4)
Surgical Right-sided craniectomy – n (%) Left-sided craniectomy – n (%) Bifrontal craniectomy – n (%)	84 (57.9) 58 (40.0) 3 (2.1)
Indication Acquired brain injury – n (%) • Haemorrhagic stroke • Ischaemic stroke • Traumatic brain injury • Primary infection • Haemorrhagic space-occupying lesion Postoperative complication – n (%)	99 (68.3) 47 (32.4) 28 (19.3) 13 (9.0) 10 (6.9) 1 (0.7) 46 (31.7)
Outcomes Mortality – n (%) Cranioplasty performed – n (%) MRS at last follow up – median (IQR) Pre-existing comorbidities – median (IQR)	45 (31.0) 39 (26.9) 5 (1–6) 1 (1–3)
Psychiatric outcomes Pre-existing psychiatric diagnosis – n (%) Any psychiatric medication prescribed – n (%) New psychiatric medication initiated post-craniectomy – n (%)	30 (20.7) 49 (33.8) 27 (18.6)

*SD* standard deviation, *IQR* interquartile range, *GCS* Glasgow Coma Scale, *ASA* American Society of Anaesthesiologists Physical Status, *IMD* Index of Multiple Deprivation, *MRS* Modified Rankin Scale

**Table 2:** Comparative statistics of predictor variables between acquired and postoperative complication groups

Variable	ABI (*n* = 99)	Postoperative complication (*n* = 46)	*p*-value
GCS – median (IQR)	9 (6–12)	15 (13–15)	< 0.0001
Age, years – mean (SD)	51.0 (13.2)	53.2 (13.8)	0.476
ASA grade – median (IQR)	4 (3–4)	3 (2–3)	< 0.0001
MRS – median (IQR)	5 (3–6)	4 (1–6)	0.013
Mortality – n (%)	33 (33.3)	12 (26.1)	0.443
Comorbidities – median (IQR)	1 (1–2)	2 (1–4)	0.506
Cranioplasty performed – n (%)	27 (27.3)	12 (26.1)	1.000
Pre-existing psychiatric diagnosis – n (%)	19 (19.2)	11 (23.9)	0.516
Any psychiatric medication prescribed – n (%)	31 (31.3)	18 (39.1)	0.451
New psychiatric medication initiated – n (%)	17 (17.2)	10 (21.7)	0.502

*ABI* Acquired Brain Injury, *GCS* Glasgow Coma Scale, *ASA* American Society of Anaesthesiologists Physical Status, *MRS* Modified Rankin Scale. Continuous variables compared using the Mann-Whitney U test; categorical variables using Fisher's exact test

**Table 3:** Multivariate logistic regression of candidate predictors for new prescription of psychiatric medication following craniectomy.

Predictor	aOR	95% CI	*p*-value
Age	1.018	0.981–1.057	0.349
GCS	1.093	0.947–1.263	0.225
Pupils	0.929	0.281–3.077	0.905
ASA	0.850	0.498–1.449	0.550
IMD	1.000	1.000–1.000	0.969
Comorbidities	0.767	0.562–1.047	0.095
ABI vs. Postoperative Complication	1.182	0.396–3.528	0.765
Laterality	1.112	0.454–2.721	0.816

n=145. *aOR* adjusted odds ratio, *CI* confidence interval, *GCS* Glasgow Coma Scale, *ASA* American Society of Anaesthesiologists Physical Status, *IMD* Index of Multiple Deprivation, Laterality (coded as left versus right/bifrontal). Multivariable AUC: 0.645

**Table 4:** LASSO selected candidate predictors (standardised coefficients) for new prescription of psychiatric medication following craniectomy

Predictor	Coefficient
Age*	+ 0.0112
GCS*	+ 0.2451
Comorbidities*	−0.1883
ASA*	-0.0137
Pupils	0
IMD	0
ABI vs. Postoperative Complication	0
Laterality	0

n=145. *GCS* Glasgow Coma Scale, *ASA* American Society of Anaesthesiologists Physical Status, *IMD* Index of Multiple Deprivation, Laterality (coded as left versus right/bifrontal)

* indicates non-zero coefficients

Selected C: 0.35938; selected λ: 2.78256

## Discussion

To the best of our knowledge, this is the first study to use patients with mixed indications for craniectomy to develop and internally validate a predictive model for new psychiatric drug initiation. Whilst no independent predictor reached statistical significance on multivariable analysis, penalised modelling retained higher GCS, lower ASA, fewer comorbidities, and age as non-zero predictors of new psychiatric drug initiation. The penalised model retained a positive coefficient for presenting GCS. This may be explained by preserved cortical function, allowing patients to report affective symptoms. Conversely, patients with poor neurological status may be under-recognised psychiatrically. Comorbidity burden was retained with a negative coefficient, which may reflect prescription bias or competing comorbidities that reduce the rate of psychiatric diagnoses. Our data suggest that psychiatric outcome is not linearly related to injury severity, and that functional scales (MRS, GCS) fail to adequately capture mood-related data. There is a clear mismatch between functional and psychiatric recovery in this group of patients.

Neuropsychiatric morbidity is highly prevalent after acquired brain injury, with diagnoses of depression, anxiety and apathy common in TBI and stroke cohorts [3, 11]. This is a major contributor to long-term disability and a significant burden on carers. In TBI patients, the relationship between injury severity and the incidence of depression is not strictly linear, with patients exhibiting time- and severity-dependent variation [10, 13]. Evidence from TBI cohort studies shows that the rates of depression may increase from mild to moderate injury severities, with some studies demonstrating higher rates of depression in milder injury groups [5]. In patients with severe injury, there may be a limited ability to recognise psychiatric symptoms due to profound cognitive dysfunction and reduced expressive capacity. The direction of the GCS coefficient is, at least in part, explained by this detection and treatment effect. Therefore, it may not reflect true prevalence, but rather the recognition and subsequent diagnosis of psychiatric disease. Moreover, our primary outcome was defined as initiation of psychiatric medication following craniectomy. This is largely influenced by clinician behaviour, with competing clinical priorities in higher severity brain injury groups alongside the challenges of polypharmacy, directly limiting diagnoses made. Counterintuitive protective associations for poor neurological baseline status (low GCS, high ASA, greater number of comorbidities) suggest that the model likely captures prognosis and clinician behaviour rather than true psychiatric morbidity. A model utilising only baseline clinical data as predictor factors will deprioritise those most vulnerable to psychiatric underdiagnosis, further highlighting the clinical importance of longitudinal psychiatric screening in all neurosurgical patients who have undergone life-changing procedures.

The literature on craniectomy survival focuses primarily on mortality and functional outcome scores. Psychiatric outcome is almost entirely absent in major craniectomy trials. However, psychiatric morbidity following craniectomy is well documented [1, 4, 21, 25]. In our cohort, just over one-third of our patients were prescribed psychiatric medications. Whilst there are likely significant psychosocial reasons for patients developing psychiatric disorders following a craniectomy, we cannot ignore the neurophysiological mechanism that may underpin this. A craniectomy alters intracranial pressure dynamics, and a skull defect alters cerebral blood flow and cerebrospinal fluid hydrodynamics. Studies have shown that the syndrome of the trephined demonstrates reversibility of cognitive and affective deficits, with an improvement in behavioural and cognitive functions following a cranioplasty [2]. This supports the potential reversibility of neuropsychological deficits with skull restoration. The period following craniectomy may, therefore, represent a neurorestorative period where neurophysiology remains modifiable. Interventions that aim to optimise intracranial compliance and cortical perfusion may alter the psychological trajectory for patients in the rehabilitation phase of their illness.

The survivor-only cohort sensitivity analysis yielded an optimism-corrected AUC of 0.582, which was almost identical to the full-cohort optimism-corrected AUC of 0.580. This suggests that mortality does not confound the relationship between baseline predictor variables and new-onset psychiatric medication prescription. Indeed, if mortality suppressed true predictive effects, we may expect that restricting analyses to only survivors would improve model discrimination; however, this did not happen. The observed consistency of findings across the full and survivor cohorts may indicate the model’s limited discriminative ability to be due to a true difficulty in predicting psychiatric drug prescriptions from baseline clinical data. It may be that psychiatric drug prescriptions following craniectomy are driven by factors beyond baseline clinical status, including premorbid psychiatric characteristics, psychosocial determinants, and prescribing culture, all of which were not factored into this retrospective chart analysis, warranting analysis in further prospective studies.

Our risk prediction model has clear limitations, with an optimism-corrected AUC of 0.580, sitting below the threshold for clinical utility. It is therefore not appropriate for clinical deployment in its current form. Although our model does not discriminate appropriately for clinical use, the frequency of new psychiatric medication initiation in this cohort highlights the importance of a robust risk stratification tool in the early detection of patients at increased risk. This would prompt clinicians to consider earlier neuropsychiatric follow-up during rehabilitation, with smoother access to psychiatric services increasing rehabilitation capability, by improving therapy engagement, and facilitating social reintegration. Moreover, psychiatric morbidity contributes to significantly high healthcare-utilisation and caregiver burden, with risk stratification tools supplementing a more targeted allocation of psychiatric resources.

The retrospective design of our study makes the model susceptible to unmeasured confounding, making it difficult to establish causality. We were also reliant on the accuracy of documentation when collecting retrospective data. We used a surrogate outcome to determine psychiatric morbidity, defined as those who started psychiatric medications following a craniectomy, and we acknowledge that prescribing psychiatric medication does not constitute a formal psychiatric diagnosis. Some psychiatric morbidity may be untreated pharmacologically and therefore missed from the data set. Formal psychiatric diagnoses were not available for all patients and clinical indications for medication prescription were not systematically documented. However, patients meeting our primary outcome criteria were primarily started on mood-active antidepressants and the majority remained on treatment through to the final review, with those who died remaining on treatment at the time of death. Further, we had a small number of primary outcome events, reducing our EPV ratio and requiring penalisation to mitigate overfitting. Therefore, associations found do not imply causation. Craniectomy size was not included as a candidate predictor, as bone flap dimensions were infrequently documented in operative notes and cranial imaging was not obtainable (due to archiving) for some patients. Retrospective estimation from imaging is also susceptible to error, and there is no consensus on the acceptable metric.

Future studies should focus on utilisation of multicentre datasets, external validation of this model, and integration of neuroimaging metrics as candidate predictors. Prospective studies would allow formal, longitudinal psychiatric data to be collected using gold-standard neuropsychological batteries. A further avenue of research is to determine the temporal relationship between cranioplasty timing and psychiatric trajectory. Correlating these outcomes with neuroimaging and molecular data, would allow us to establish if analyses of network connectivity or biomarkers of cortical recovery are meaningful predictors.

## Conclusion

Psychiatric morbidity is an under-recognised risk for patients that survive a craniectomy. Neurosurgical scales capture outcomes of physical disability well but neglect psychological outcomes, with complex biopsychosocial risks absent from routine perioperative variables. We propose that risk stratification at the time of craniectomy requires further investigation, as our findings indicate that baseline clinical variables alone are insufficient. The integration of neuropsychiatric testing into routine clinical pathways may optimise rehabilitation potential and aid a more holistic recovery.

## Data Availability

The study was a retrospective clinical audit conducted within an NHS trust, and the dataset contains confidential, potentially identifiable patient information that cannot be shared under NHS information governance policies.
